# Brexit and Animal Protection: Legal and Political Context and a Framework to Assess Impacts on Animal Welfare

**DOI:** 10.3390/ani8110213

**Published:** 2018-11-18

**Authors:** Steven P. McCulloch

**Affiliations:** Department of Politics and Society, University of Winchester, Winchester SO22 4NR, UK; Steven.McCulloch@winchester.ac.uk; Tel.: +44-(0)1962-827-465

**Keywords:** Animal health, animal welfare, animal protection, Brexit, Common Agricultural Policy, Conservative Party, European Union, World Trade Organisation

## Abstract

**Simple Summary:**

The British public voted to leave the European Union (EU) in a 2016 referendum and the United Kingdom (UK) is set to leave in March 2019. The UK has been part of the EU or the European Communities (EC) before it for around 50 years. Britain has had a major impact on EU animal protection laws and the UK as a member state has been substantially influenced by EU law. Brexit represents a major political change that will affect animal protection in the UK, the EU and internationally. Given the far greater numbers of animals used in agriculture, the impact of the UK’s departure from the EU on farm animals will determine whether Brexit is overall good or bad for animal protection. A major threat that Brexit poses is importing lower welfare meat and dairy products to the UK. A major opportunity post-Brexit is reform of agricultural policy to reward high animal welfare outside of the EU Common Agricultural Policy (CAP). A soft Brexit, where the UK remains aligned to the EU in policy and trade, reduces the risks Brexit poses to animal protection. A hard Brexit means major threats to animal welfare are more likely to materialise. Further research is required to investigate whether the various threats and opportunities are likely to materialise and whether Brexit will be, all things considered, good or bad for animal protection.

**Abstract:**

The British people voted to leave the European Union (EU) in a 2016 referendum. The United Kingdom (UK) has been a member of the EU since the Maastricht Treaty was signed in 1993 and before that a member of the European Communities (EC) since 1973. EU animal health and welfare regulations and directives have had a major impact on UK animal protection policy. Similarly, the UK has had a substantial impact on EU animal protection. Brexit represents a substantial political upheaval for animal protection policy, with the potential to impact animal welfare in the UK, EU and internationally. Brexit’s impact on farmed animals will determine the overall impact of Brexit on animals. A major threat to animal welfare is from importing lower welfare products. A major opportunity is reform of UK agricultural policy to reward high welfare outside the Common Agricultural Policy (CAP). A soft Brexit, in which the UK remains in the single market and/or customs union, mitigates the threat of importing lower welfare products. A harder Brexit means threats to animal welfare are more likely to materialise. Whether threats and opportunities do materialise will depend on political considerations including decisions of key political actors. The Conservative Government delivering Brexit has a problematic relationship with animal protection. Furthermore, Brexit represents a shift to the political right, which is not associated with progressive animal protection. There is significant political support in the Conservative Party for a hard Brexit. Further research is required to investigate whether the various threats and opportunities are likely to materialise.

## 1. Introduction

The British people voted in a 2016 referendum to leave the European Union (EU). The United Kingdom (UK) has been a member of the EU since the Maastricht Treaty was signed in 1993 and before that a member of the European Communities (EC) since 1973. EU regulation has had a major influence on UK animal health and welfare policy. Similarly, as one of the larger members of the EU and with a history as a leader in animal welfare, the UK has had a substantial influence on EU animal protection policy [1].

The European Union (EU) is a political and economic union composed of 28 member states. The total human population in the EU is around 510 million. Based on its size and economic importance, the EU has been described as an emerging superpower [2]. Its political institutions are the European Council, European Commission, Council of the European Union (Council of Ministers), European Parliament and the Court of Justice of the EU. The EU has an internal single market without borders of goods, services, labour and capital to promote free trade and economic prosperity. The EU is also a customs union and all 28 member states form an effective single territory for customs purposes [3].

Independent economic analyses have forecast that Brexit will have a negative economic impact in the medium term. The International Monetary Fund (IMF) has reported that Brexit has been associated with a weakening of Sterling, reduced business investment and lower growth [4]. The government’s own impact assessments have shown that Brexit will make the UK worse off in all scenarios. These scenarios include a Norway-style agreement that provides continued access to the EU single market, a Canada-style free trade agreement and a ‘no deal’ outcome in which the UK trades with the EU on World Trade Organisation (WTO) terms [5,6]. Over a 15 year timeframe, GDP will be two per cent lower in the Norway model, five per cent lower in the Canada free trade model and eight per cent lower trading on WTO rules [7].

In Theresa May’s Chequers 2018 agreement, the government put forward a policy that was effectively a soft Brexit for agriculture. The government proposed that goods, including agricultural goods, would remain in alignment with the EU’s rules – essentially following the single market [8]. This would mean that the UK would continue regulatory alignment with the EU, as well as applying the same external tariffs to agricultural imports in a facilitated customs union for agricultural products [9].

In the context of animal protection, a number of reports have been published on the potential impacts of Brexit [1,10,11,12]. This paper reviews the threats and opportunities discussed in these reports. The paper then discusses the EU/UK animal protection regulatory landscape and the political context of Brexit. The aim of the paper is to provide crucial regulatory and political context for future research on how likely threats posed by and opportunities presented by Brexit are likely to materialise. That future research will address the following question: Will Brexit, all things considered, be good or bad for animal protection? This paper provides a framework and explores salient factors to facilitate investigating this fundamental question in future research.

This paper is structured in nine sections. Section 2 provides two vignette arguments from opposing sides on the impact of Brexit on animal protection. Section 3 describes a framework for assessing how Brexit is likely to impact animal protection. Section 4 discusses what categories of animals will be most affected by Brexit. Section 5 reviews some of the potential impacts of Brexit on animal protection discussed in various published reports. Section 6 describes the legal context of Brexit and animal protection. How are the EU and UK animal health and welfare regulatory landscapes related? Section 7 discusses the political context of Brexit. What are the political forces that have motivated and continue to shape Brexit? The political context is crucially important as the overall impact of Brexit on animal protection will be influenced, if not determined, by the various political developments in play. Section 8 discusses how different forms of Brexit—soft, hard and WTO rules—will impact animal protection. Finally, Section 9 discusses how policy making in a nation within a political union such as the EU differs from that in a nation outside of a political union, in the context of animal protection.

## 2. Brexit and Animal Protection: Vignette Arguments for and Against

To provide context for further discussion, especially for readers outside of the EU and UK, this section includes two opposing vignette characterisations of the Brexiteer case to leave the EU and the Remainer case to stay in the union. These sections are characterisations of Brexiteer and Remainer arguments and further analysis is required to investigate the validity of the various claims.

### 2.1. The Animal Protection Case for Leaving the European Union

*The UK is a world leader in animal welfare. She is held back in her ambitions by being part of the EU. For instance, the EU prevents the UK from banning live animal exports. Similarly, membership of the EU means the UK cannot ban the importation and sale of foie gras, the production of which is effectively banned in the UK. When the UK leaves the EU, she will be free to ban live animal transport and the importation and sale of foie gras.*

*The Common Agricultural Policy (CAP) of the EU supports intensive farmers and large landowners. When the UK leaves the EU, she will be able to reward farmers for high animal welfare and export her high quality produce to the rest of the world. The UK will be free to independently trade with traditional allies like the US and Australia. The UK government has stated it will protect animal welfare standards after Brexit. If the UK does import US and other nation’s products as part of a trade deal, British consumers will be free to not purchase them, if they wish. Regardless, if chlorinated chicken is good enough for Americans, it should be good enough for Brits too.*

*EU membership means that the UK cannot control her own borders and unscrupulous pet smugglers from Eastern European member states transport thousands of sick puppies and kittens into Britain each year. When the UK leaves the EU she will be able to make laws to clamp down on this practice.*

### 2.2. The Animal Protection Case for Remaining in the European Union

*The UK is a world leader in animal welfare. She has used her position in the EU to influence animal protection policy in the largest trading bloc in the world. For instance, the UK was the driving force behind Article 13 of the Treaty of Lisbon, which recognises animals as sentient beings. Based on the Treaty of Lisbon, the EU and member states must pay full regard to the welfare of animals when formulating and implementing policy.*

*The EU has some of the highest welfare standards in the world. The EU has banned barren battery cages for laying hens, calf crates for veal production and limits the use of sow stalls for pregnant pigs. Member states are free to implement even higher welfare standards, which the UK has in many cases taken advantage of. The EU prohibits hormone-treated beef, chlorine-washed chicken and growth promoters in pigs. The EU has fought trade battles with the US to prevent these products from entering the single market because they are associated with lower food safety and animal welfare standards.*

*Outside of the EU, the US—with its powerful agricultural lobby—will force the UK to import US goods produced with far lower welfare and food safety standards. Where there is doubt the EU protects its citizens by applying the precautionary principle, whereas the US supports large agri-business. Chlorinated chicken will swamp British supermarket shelves. Brits will be consuming hormone-treated beef for their Sunday dinner.*

*Finally, the EU has banned the importation and sale of dog, cat and seal fur based on public morality. It has some of the most stringent regulation on animal experimentation in the world. The EU has prohibited the testing of cosmetics and ingredients on animals and their import and sale, as this practice is impossible to justify on moral grounds. It funds an agency that shares data to avoid the unnecessary duplication of animal experiments for medical and toxicological research.*

These vignettes are useful to provide some context on the impact of Brexit on animal protection in the UK and further afield. The passages also hints at how the polarised debate on Brexit permits opposing sides to make claims related to animal protection to serve their purposes. For this reason, in order to investigate such claims, it is important to use a framework to assess them. The following section provides such a framework.

## 3. A Framework to Investigate the Impact of Brexit on Animal Welfare

Brexit is an enormous political upheaval between the UK and the EU. There are various categories of animals and species at play. Brexit will affect animals in the UK, in the EU27 and in future trading nations and blocs. There are substantial political and legal uncertainties. The following questions provide a comprehensive framework for assessing the impact of Brexit on animals:

1. What is the current relationship between the UK and the EU in animal health and welfare policy, that is, what is the status quo?
2. What is the political context of Brexit, that is, what are the political considerations that are likely to determine the impact of Brexit on animal protection?
3. What are the threats and opportunities to animal protection of Brexit?
4. What are the threats and opportunities to animal protection of Brexit to different categories of animals?
5. What are the threats and opportunities to animal protection of different forms of Brexit?
6. What are the threats and opportunities to animal protection of Brexit geographically, that is, in the UK, the EU and internationally?
7. What are the magnitude of the various threats and opportunities of Brexit?
8. How likely are the various threats and opportunities of Brexit to animal protection to materialise?
9. All things considered, will Brexit be a net positive or negative for animal protection in the UK, EU and internationally?

How Brexit impacts animal welfare will be influenced by a number of factors that are complex and interrelated. Some of these factors will be related to and under the control of the UK government and Parliament; others will not. Broadly, these factors include first, the form of Brexit which the UK and EU conclude. Whether the UK and EU conclude a hard or a soft Brexit, for instance, will have a very substantial impact on animal welfare. Secondly, the political nature of the UK government and Parliament, now and in the medium term, will have a substantial impact on animal protection. Thirdly, EU policy toward the UK during and after the Brexit negotiations will have a major impact on animal protection. Fourthly, the policy of third countries such as the US, during Brexit negotiations and when negotiating free trade agreements, will have a major impact on animal protection. Fifthly, in addition to these factors, there are additional uncertainties such as how WTO rules on animal protection will be interpreted going forward.

The discussion in the remainder of this paper focuses on questions 1–5 in the framework above. The paper discusses the legal and political context of Brexit. It describes how soft and hard forms of Brexit might impact animal protection. It also reviews threats posed and opportunities presented by Brexit discussed in a number of reports on the subject. Before discussing the legal and political context of Brexit, the next section of the paper aims to provide some focus based on the categories of animals that are more likely to be affected, positively or negatively, by Brexit. If, for instance, the impact on one particular category of animal is far greater than that on the other categories of animals, then we can focus our attention on that category.

## 4. What Categories of Animals will be Most Affected by Brexit?

For the sake of convenience, I have adopted the following classification in accordance with human use of, or relationship with, animals: (i) animals in farming, (ii) animals in research, (iii) wild animals and (iv) companion animals (including equines). Table 1 below illustrates populations of each category of animal in the UK, EU and US. The US is included for illustration of a large nation outside of the EU which Brexit provides the potential for a change in the UK’s trading relationship.

Table 1 reveals that there are far larger numbers of farm animals across the UK, the EU and the US. In the UK, around one billion land animals are raised and slaughtered in each year [14]. In contrast, there are around four million experimental procedures performed annually on laboratory animals in the UK [17]. In the UK there are around 51 million pet fish, 9 million pet dogs and 8 million pet cats [22]. Finally, there are around one million equines [23]. To put these figures in context, the human population in the UK is around 66 million [24]. 

The numbers of animals in each of these categories are very substantial. We should remember that each of these animals is an individual sentient life. Government policy has the potential to affect the lives of all categories of animals [25,26]. However, for present purposes, the reality that farm animals vastly outnumber other categories means that how Brexit affects farm animals is very likely to determine whether Brexit has a net positive or negative impact on all animals. Matheny and Leahy describe the consequences of numbers on animal protection policy vividly in the US context:

Farm animals represent ninety-eight percent of the animals raised and killed in the United States. Compared to farm animals, the number of animals hunted, kept as companions, used in labs, reared for the fur industry, raced and used in zoos and circuses is insignificant. The “animal-welfare issue” is thus numerically reducible to the “farm-animal-welfare issue”.

(p. 326) [15]

Of course, despite these numbers, it might be the case that Brexit disproportionately impacts not farm animals but wild, experimental or companion animals. This scenario, however, would be extremely unlikely. Indeed, Brexit will impact farm animals as a category more than other categories of animals. EU member states trade in the internal single market. To enable a level playing field, EU member states have substantial regulatory alignment in agriculture [10,27]. The Common Agriculture Policy (CAP), as its name suggests, is common to all EU member states. Brexit means the UK breaking away from the EU, being outside of the CAP and potentially outside of the single market. Thus, Brexit is likely to lead to divergence in agricultural policy and possibly trading relationships, between the UK and EU. Given these points, the impact of Brexit on animal protection will be reducible to its impact on farm animals in the UK, EU and internationally.

The second major point to make based on the numbers in Table 1 relates to the relative numbers of animals in the UK, the EU and the US. There are far more farm animals raised and slaughtered in the US (10 billion) compared to the EU (4.7 billion) and the UK (1 billion). These numbers suggest that the overall impact of Brexit on animal protection may not be determined by its effect on animals in the UK but on animals in the EU and internationally. This is an important point, because in considering how Brexit will impact animal protection all things considered, there is a risk that one will have an undue focus on animals in the UK and perhaps the EU.

The next section reviews some of the key findings of authoritative reports on Brexit and animal protection.

## 5. Brexit Impacts on Animal Protection: Threats and Opportunities

*Brexit: getting the best deal for animals* is a wide-ranging report published by the Wildlife and Countryside Link (Link) and the UK Centre for Animal Law (A-Law). The report is a product of a large coalition of leading UK animal protection NGOs including the Royal Society for the Prevention of Cruelty to Animals (RSPCA), Compassion in World Farming (CIWF), World Animal Protection (WAP) and the International Fund for Animal Welfare (IFAW). The report describes how Brexit presents a major juncture in the history of UK animal protection:

As the UK prepares to leave the EU, the welfare of animals is at a critical crossroads and selecting the route ahead will determine the welfare of billions of animals. We have a once in a lifetime opportunity to either define or undermine our country’s identity and reputation as a global leader in animal welfare science and standards.

(p. 5) [1]

The report is authoritative and details potential reforms for the four major categories of animals: farmed, wildlife, research and companion. The report does not, however, assess the probability of the various opportunities and threats actually materialising. Rather, the report has been written after the referendum vote in 2016, with a view to maximising the benefits for animals. This quotation also highlights how many in the animal protection community view Brexit as an opportunity in a broad sense to campaign for reforms in animal protection policy. Thus, many of the opportunities discussed in the reports are not related to policy that could not be made before Brexit, with the UK as a member state of the EU. Rather, Brexit as a major political event is considered as an opportunity for reform.

In the section on Animals in Agriculture the report states:

Two factors will be decisive in determining the post-Brexit level of animal welfare: trade issues and the arrangements for farm support payments that replace the Common Agricultural Policy (CAP).

(p. 24) [1]

The authors writing these words are referring to animals in agriculture. Despite this, given that the impact of Brexit on animal protection is reducible to its impact on farm animals, the above statement is arguably applicable to animal welfare per se. Trade issues and arrangements for farm support payments are therefore critical to focus on when assessing whether Brexit is likely to have net positive or negative impacts for animal protection.

*Brexit and animals: Opportunities and threats, UK animal welfare under different models of relations with the European Union* (EU) is a report produced by a smaller number of animal protection NGOs, including the RSPCA, CIWF and WAP. The report very usefully sets out the status quo on UK farm animal protection as an EU member state:

The arrangement the UK currently has inside the EU is a high level of animal welfare standards protected by external tariffs. This should be retained in any future trade arrangement the UK will have with the EU and with other trade partners, to prevent the race to the bottom that could arise from a surge in imports of products produced to lower animal welfare standards.

(p. 4) [11]

This concise description of the status quo is elaborated on later in this paper. The situation of the UK as a member of the EU with high tariffs preventing the importation of agri-goods produced in lower welfare standards is a fundamental baseline to judge how Brexit might impact animal welfare. 

A 2017 House of Lords report *Brexit: farm animal welfare* describes farm animal welfare standards in the UK and the broad stakeholder support to retain these after Brexit:

The UK has some of the highest farm animal welfare standards in the world. UK producers are rightly proud of these standards and there is cross-sector support for maintaining high levels of farm animal welfare after Brexit.

(p. 3) [10]

The Taskforce report assesses animal welfare under different models of Brexit. It assesses the following models of UK-EU relations: (i) continued EU membership; (ii) European Economic Area (EEA), or the ‘Norway model’; (iii) the ‘Swiss model’; (iv) continued membership of the EU customs union; (v) a deep and comprehensive free trade agreement (DCFTA); and (vi) WTO rules. Table 2 below is reproduced with permission from the Brexit and Animals Taskforce. 

The animal welfare issues included in the table are formulated such that a tick is generally positive for animal protection. For instance, banning live exports for fattening and slaughter under the ‘Swiss model,’ the DCFTA or under WTO rules is positive for animal protection. In contrast, a cross is generally negative for animal protection. For instance, continued membership of the EU, the ‘Norway model’ or the ‘Swiss model’ all preclude the possibility of method of production labelling. Method of production labelling is supported by animal protection NGOs because it provides information for consumers that is likely to reduce the purchase and consumption of lower welfare products and increase that of higher welfare products.

The post-Brexit model of the UK-EU relationship in some cases has little or no impact on animal protection. For instance, Table 2 illustrates that all models permit continued participation in the PETS scheme. The Pet Travel Scheme (PETS) was introduced to permit the travel of pet dogs, cats and ferrets within the EU. It has since expanded to include non-EU nations, which is why the UK would remain a member of PETS under the various post-Brexit models in Table 2. Prior to PETS, the UK had a compulsory six month quarantine for imported pet dogs and cats. The quarantine period was considered to cause welfare problems and the PETS scheme a successful reform. However, if the UK does not successfully negotiate a withdrawal agreement with the EU, the UK will drop out of the EU without any deal. This would mean that the UK membership of PETS would be jeopardised. The UK Government is seeking discussions with the EU on the issue and has published technical guidance on this possibility [28].

The impact of Brexit on animal protection can be considered in two ways. First, there is the nature of the divorce between the UK and the EU and the deal or agreement on the future relationship. Secondly, there are trade deals that the UK will negotiate, both with the EU27 and with non-EU nations such as the US. As discussed later in this paper, these two considerations are related but distinct. Related to this distinction, in the context of Table 2, the Brexit and Animals Taskforce authors state the following:

The UK does not lose or gain an ability to defend trade restriction based on animal welfare or to promote better welfare in partner countries based on each scenario. However, this ability might seriously decrease with the loss of the access to the EU market as leverage particularly for farm animals. This could have a very detrimental long-term impact on animal welfare in the UK… but it cannot be represented in the table.

(p. 10) [11]

Given the impact of Brexit on animal protection will be reducible to its effect on farm animals, this is a major point. Furthermore, trade deals will influence animal protection on an international basis, which means potentially impacting a far greater number of animals. Hence, access to the EU market as leverage will be critical to how Brexit impacts animal protection.

The Brexit and Animals Taskforce also report how leaving the EU affects the UK’s access to key animal protection agencies. Under all scenarios aside from remaining a member state of the EU, the UK will no longer have full access to the Trade Control and Expert System (TRACES). TRACES is a system used by EU member states to monitor the movement of animals commercially into and within the union. TRACES also underpins the Tripartite Agreement regulating trade in horses between the UK, France and Ireland. The loss of TRACES may lead to a significant increase in checks at borders [11]. Increased checks take significantly more time and could lead to a substantial worsening of welfare for farm animals and equines.

Finally, Peter Stevenson has published *A better Brexit for farm animals: What the Government must do to protect welfare standards* [12]. Stevenson is legal advisor to CIWF and has written a number of reports on UK and EU regulation, trade and related matter such as the WTO and animal protection. In his report, he lists the following as key factors that will determine how Brexit impacts animal welfare:

- Trade
- Agricultural subsidies
- Consumer support for higher welfare products based on labelling by method of production
- Public procurement (e.g. the National Health Service procuring high welfare food)
- Continuing political commitment to animal welfare

Early in his report, Stevenson summarises how the UK government’s stated political ideals may be difficult to realise in the real world:

A Written Ministerial Statement refers to the Government’s ambition to “set a global gold standard for animal welfare as we leave the EU.” However, future trade deals may make these ambitions very difficult to realise in practice.

(p. 6) [12]

These four reports are well researched and are either informed or written by leading experts. They provide an indispensable resource on the potential impacts of Brexit on animal welfare. This paper is informed by these reports but seeks to go beyond the reports by providing a firm basis to investigate whether, all things considered, Brexit *will* be positive or negative for animal protection. How likely is it that the various threats and opportunities discussed in the reports cited above will materialise? This question is informed by the legal context of the EU and UK regulatory landscape, as well as the political context of Brexit. Section 6 discusses the EU and UK animal health and welfare regulatory landscape.

## 6. EU Animal Health and Welfare Regulation

The UK as a Member State of the EU: The Status Quo and Animal Welfare

The UK has been a member of the EU or its precursor the European Economic Community (EEC) and EC for around fifty years. EU and UK animal protection regulation, especially on farmed animals, has co-evolved during the last 43 years, when the first farm animal welfare regulation was passed. Around 80 per cent of UK animal protection regulation is based on EU laws [29,30]. Of the 44 EU animal welfare laws, 31 in the acquis communautaire are regulations and decisions and 13 are directives. The European Union (Withdrawal) Act 2018 has nationalised the great majority of this law, although there are concerns about the powers the Act gives to ministers to amend legislation where relevant to make it appropriate after Brexit [31].

As an EU member state, regulations and decisions are applicable to UK law without national implementation. For instance, Regulation (EC) No. 1/2005 on the protection of animals during transport and related operations was directly applicable. Directives are implemented by member states to satisfy the policy objectives of the EU directive. For instance Directive 2008/120/EC laying down minimum standards for the protection of pigs is transposed into English law via The Welfare of Farmed Animals (England) Regulations 2007 and The Mutilations (Permitted Procedures) (England) Regulations 2007. Since animal health and welfare policy is a devolved responsibility in the UK, similar regulations are in place in Scotland, Wales and Northern Ireland.

The UK has been a beacon for animal protection in the EU, being instrumental to a progressive culture for animal welfare policy and providing the political impetus for reform. In effect, the UK has leveraged its progressive animal welfare stance to benefit a much larger community of currently 510 million people [11,32]. For instance, the UK was instrumental in lobbying for Article 13 of the Treaty of Lisbon. Article 13 recognises animals as sentient and mandates that the EU and member states must pay full regard to animal welfare in formulating and implementing policy in certain policy areas [33].

On a global basis, the EU has very high welfare standards relative to other nations. The EU has general farm animal welfare legislation for animals on farm, in transport and at slaughter. It has species-specific legislation for veal calves, pigs, broiler chickens and laying hens. In most areas of on-farm animal welfare policy, EU member states are free to implement more stringent animal welfare laws. Indeed, the UK and some other member states have on a number of occasions transposed EU directives into national laws in terms that are more stringent than those mandated by the EU. Table 3 outlines some key EU and UK animal protection regulation for the different categories of animals.

There is substantial trade in live animals and animal products between the UK and the EU. The UK is the largest export market for animal products of the EU27 (The EU27 refers to the 28 EU member states minus the UK). Similarly, the EU27 is the largest export market for animal products of the UK. The UK imports around Euros 11 billion in livestock products from the EU and exports around Euros 5.2 billion. Hence, the UK and EU27 currently have a very substantial trade relationship in animal products, with the EU27 having a significant trade surplus with the UK [32].

The EU largely prevents the importation and sale of animal products produced with lower food safety and animal welfare standards by setting high tariffs to protect its farmers and prohibiting through legislation imports of products such as beef from cattle injected by hormones that are illegal in the EU. Article 12 of Council Regulation 1099/2009 provides that imported meat products need to have been from animals slaughtered to welfare standards that are at least equivalent to those of the EU. Regulation (EC) No 1523/2007 prohibits the importation and sale of products which contain dog or cat fur. EU policy restricts the importation of certain other products: Regulation (EC) No 853/2004 of the European Parliament and of the Council of 29 April 2004 laying down specific hygiene rules for food of animal origin prohibits chlorine-washed chicken; Directive 96/22/EC as amended by Directive 2003/74/EC prohibits hormone-treated beef; and EC Directive 96/22/EC as amended by Directive 2003/74/EC prohibits ractopamine pork. These are common agricultural practices in the US, Australia and New Zealand. However, it is high tariffs that prevent the importation of most animal products. For instance, the EU has tariffs of over 40% for pig meat and 50% for sheep and dairy products. Without such high tariffs, the EU would be flooded with animal products produced with far lower animal welfare and food safety standards [30].

In other policy areas, the EU has progressive animal welfare regulation. For instance, Directive 2010/63/EU of the European Parliament and of the Council of 22 September 2010 on the protection of animals used for scientific purposes prevents duplication of animal testing. The European Union Reference Laboratory for Alternatives to Animal Testing (EURL-ECVAM) is the EU agency that helps to implement the Directive 2010/63/EU. EU law also prohibits the testing of cosmetic products or ingredients on animals, as well as banning the importation and sale of the same.

## 7. Brexit: The Political Context and Animal Welfare

### 7.1. The UK Referendum and Brexit

The 2016 UK referendum on EU membership was in large part motivated by an internal Conservative Party issue. Since the 1990s, the Conservative Party has been divided on Europe, with a significant right wing Eurosceptic faction [34]. Concerned about the growth of the United Kingdom Independence Party (UKIP) and wanting to settle the Europe question, the Prime Minister David Cameron held a referendum in 2016. Boris Johnson and Michael Gove, both on the right of the Conservative Party, campaigned for Vote Leave [34,35]. Vote Leave won by 51.9% against Britain Stronger In Europe’s 48.1%. Cameron had campaigned to remain in the EU. When the result was announced, he announced his resignation and, later that year, Theresa May became Prime Minister. Despite May campaigning to remain, she appointed several right wing Eurosceptics to Cabinet to deliver Brexit. These included Boris Johnson (Foreign Office), Liam Fox (International Trade) and David Davis (Secretary for exiting the EU) [36]. Michael Gove later re-entered Cabinet as Department for Environment, Food and Rural Affairs (Defra) Secretary in 2017 [37].

Theresa May called an earlier than expected general election in 2017, with the objective of increasing her working majority of 17 seats in Parliament to give her government leverage on Brexit. Despite this aim, the Conservative Party lost 13 seats. Both the Conservatives and Labour, the two largest parties in UK politics, had manifesto pledges to leave the single market and the customs union. The Liberal Democrats and the Green Party had pledged to hold a second referendum on EU membership. At the time of writing (November 2018), Theresa May is the Prime Minister of a minority Conservative Government. The Conservative Party is supported in a confidence and supply arrangement by 10 Democratic Ulster Unionist (DUP) Members of Parliament (MPs). Jeremy Corbyn leads the Labour Party, which forms the Official Opposition. Corbyn’s Labour Party has effectively supported the government to deliver Brexit, though its policy is for a softer Brexit that maintains regulatory alignment with the EU to protect workers and the environment [38].

### 7.2. British Politics and Animal Welfare

Of the two main parties, it is the centre-left Labour Party that is generally identified with more progressive animal protection policy [39,40,41]. Chaney, for instance, writes: ‘The Left exhibits a greater general propensity to advance animal welfare measures, whilst the Right is more reliant on the actions of individual MPs’ [41]. In line with this, the 2000–2005 Labour Government passed the Hunting Act 2004, which effectively outlaws foxhunting. The 2005−2010 Labour Government passed the Animal Welfare Act 2006, which advanced animal welfare from an anti-cruelty to a welfare-based paradigm [42]. In 2018 the Labour Party has published a radical 50 point plan to reform animal protection across the board in the UK. The plan includes measures such as enshrining animal sentience in law, strengthening the Hunting Act, mandatory method of production labelling of meat products and ending the badger cull [43].

In contrast, the Conservative Party has a problematic relationship with animal protection policy. For instance, the Conservative leadership pledged a free vote on the fox hunting issue in the 2017 general election campaign [44]. Theresa May publicly supported a vote to repeal the Hunting Act, despite the Act being supported by a large majority of the British public [36]. Similarly, the Conservatives have maintained a strong badger culling stance under both David Cameron and Theresa May. Badgers are culled to prevent bovine tuberculosis infection in cattle, which causes economic losses to government and the farming industry. However, independent experts, such as the Independent Scientific Group, recommended against badger culling based on a decade long field trial [45]. The badger culling policy is highly contentious in the UK and the Conservative Party and the National Farmers Union (NFU) have been heavily criticised for supporting it [46].

What of the current Conservative Government, which if it remains in power will have substantial influence on animal protection policy during Brexit? In late 2017 the Conservative Party launched a pro animal welfare publicity campaign [47]. This was motivated largely by two events. First, the Conservative Party pledge in its 2017 manifesto to have a free vote on repealing the Hunting Act 2014 was widely criticised; some argued that it contributed to May losing her majority in Parliament [36]. Secondly, the Conservative Government came under intense scrutiny in the media in late 2017 for an animal welfare issue directly related to the EU.

### 7.3. Article 13 of the Treaty of Lisbon and UK Animal Sentience Policy

In November 2017 the Conservative Government was hit by a media furore on the issue of animal sentience. The European Union (Withdrawal) Act 2018 repeals the European Communities Act 1972, which led to the UK formally joining the EC in 1973. At the same time, it nationalises EU laws onto the UK statute. EU regulations on animal welfare policy, such as on transport and slaughter, are therefore transposed into UK legislation. (EU directives were implemented onto the UK statute at the time they were passed).

Article 13 of the Treaty of Lisbon recognises that animals are sentient and mandates member states to pay full regard to animal welfare when formulating and implementing policy [33]. In contrast to EU regulations, Article 13 is part of the Treaty on the Functioning of the EU (TFEU) and so would not be transposed in the European Union (Withdrawal) Bill. For this reason, Caroline Lucas, the sole Green Party MP, tabled an amendment to incorporate Article 13 to the European Union (Withdrawal) Bill during its passage through Parliament. The Conservative Party in Parliament were whipped to vote against the amendment and the government carried the vote [48]. (Government whips are party officials that enforce party discipline by providing voting instructions to Parliamentarians.)

The Conservative Party position on the Lucas amendment caused substantial public controversy and was widely reported in the media. The government argued that Article 13 is unnecessary, since UK laws, such as the Animal Welfare Act 2006, recognise animals as sentient. However, the government’s position was roundly criticised by animal protection groups [49]. The Animal Welfare Act, for instance, is not concerned with wild animals, whereas Article 13 covers all categories of animals. Furthermore, Article 13 confers a duty on government to pay full regard to animal welfare when formulating and implementing policy. This is an important higher order protection that animal welfare laws in the UK do not prescribe [42,50].

Ultimately, the government published its own draft Animal Welfare (Sentencing and Recognition of Sentience) Bill. This, in turn, was roundly criticised, including by the Environment, Food and Rural Affairs Select Committee (EFRA Committee) for being ‘not properly thought through’ [51,52]. More importantly, the draft Bill substantially watered down obligations under Article 13. For instance, whereas Article 13 states that member states must pay ‘full regard’ to animal welfare, the draft Bill contains only ‘regard’ and in the related consultation on the Bill the government asked for input on the level of regard. Further, whereas the duty in Article 13 is conferred on the state broadly, the duty in the government bill is restricted to ‘Ministers of the Crown’ [53]. In August 2018, the government published a response to the consultation on the Animal Welfare (Sentencing and Recognition of Sentience) Bill. The government advised in its response that it will continue to consult with stakeholders on its approach to sentience and ensure that sentience is recognised after the UK leaves the EU [54].

### 7.4. Spotlight on the Conservative Government and Animal Welfare

The foxhunting pledge in the manifesto and the furore related to rejecting the sentience amendment meant that the Conservative government was under the spotlight. From an electoral perspective, animal protection is an increasingly salient policy issue, especially amongst younger voters [41,55]. Theresa May, as Chairwoman of the party in 2002, had spoken of the need for the Conservatives to rid themselves of the image of being the ‘nasty party’ [56]. In late 2017, the Conservative Government launched a media campaign to improve the Conservative Party image on animal welfare. Conservative MPs were called into Ten Downing Street, the Prime Minister’s office, to be briefed by her chief of staff on the party’s political narrative. There was an emphasis on the Conservatives being caring about the environment, animal welfare and social justice [47]. 

The Defra Secretary Michael Gove is a leading Brexiteer in government. Aside from animal experimentation and food labelling, Defra has responsibility for all animal health and welfare policy, including farm animals. As a leading Brexiteer now at the helm of the UK Government’s food and farming policy, Michael Gove has a considerable stake and influence in whether Brexit goes well for animal protection. Michael Gove is a politician that divides opinion. As Education Secretary he was a controversial figure for his policy of free schools and faced down opposition from the educational establishment [57]. At Defra, Gove has made very positive policy statements on animal protection. He has launched consultations on a raft of animal protection and environmental issues [58,59,60]. Indeed, he has published so many consultations at Defra that he has been dubbed the ‘Secretary of State for Consultations’ by his opposite number Sue Hayman [61]. Hayman was insinuating that Gove was launching consultations to defer genuine animal protection reforms and placate lobby groups. Nevertheless, Gove was awarded the ‘Politician of the Year’ by the RSPCA for his support for animal welfare policies [62]. The RSPCA was criticised for its award to Gove, in part related to his rolling out the controversial badger cull and in part because some simply did not trust the Defra Secretary on animal protection [63,64].

However, during 2017–2018 the Conservative Government has made some genuinely progressive animal welfare policy. After being criticised for omitting a pledge to ban the sale of ivory in the UK, Theresa May’s government has committed to passing legislation to effectively prohibit the trade. Boris Johnson, then Foreign Secretary and touted as a future leader of the Conservative Party, made preventing poaching elephants in Africa one of his key policies whilst in office [65]. The Mandatory Use of Closed Circuit Television in Slaughterhouses (England) Regulations 2018 makes it a legal requirement for CCTV to be used in all abattoirs in England. The passing of the law on CCTV follows a number of exposés of slaughterhouse workers abusing animals [66]. Based on the Lucy’s Law campaign [67], the government has committed to banning the commercial third-party sale of puppies and kittens in England. Those who sell puppies and kittens must now hold a licence under the Animal Welfare (Licensing of Activities Involving Animals) (England) Regulations 2018.

Wookey has discussed the opportunities and threats Brexit offers for animal welfare in the UK. He argues that the potential opportunities Brexit presents should be viewed with ‘scepticism and scrutiny’ [68]. He offers three reasons for this: First, the transposition of EU animal protection law to the UK will not be simple. Secondly, he argues that the Conservative Government position on some animal welfare issues ‘undermines its credibility’ on positive policy statements it has made on Brexit and animal welfare. Thirdly, he notes that animal welfare must be balanced with other interests *(p. 29)* [68]. Ultimately, Wookey argues that we should scrutinise the Conservative Government’s rhetoric on animal protection and replace any ‘unearned optimism’ with a sense of ‘critical scepticism’ *(p. 47)* [68].

### 7.5. Post-Brexit Trade Policy: Donald Trump’s Intervention

At the time of writing, the UK has not completed a divorce agreement with the EU. As a member state of the EU, the UK is not able to negotiate free trade agreements with third countries until it formally leaves the EU [69]. The EU27 has held firm to this position and prevented its member states from negotiating with the UK. Despite this, informal negotiations, or at least a public conversation, have started. For instance, the US President, Donald Trump, was publicly critical of Theresa May’s Chequers agreement, her government policy on Brexit, during his 2018 visit to the UK. Trump criticised May’s plan in a much publicised tabloid newspaper interview [70]. Trump’s position was motivated in part because it would prevent the US exporting its agricultural products, such as hormone-beef, to the UK [71].

A major issue relating to post-Brexit trade policy for the UK is that US and EU agricultural policy is not aligned. EU regulation is based on higher food safety and animal welfare standards [72]. In areas of significant doubt, the EU employs the precautionary principle. Indeed, the precautionary principle is a core principle in EU environmental law, enshrined in Article 191(2) of the Treaty on the Functioning of the EU [73]. In contrast, the US is a long way behind the EU in terms of animal welfare [15]. In food safety, the US does not apply the precautionary principle and puts the burden of proof squarely on those with concerns about, for instance, hormone-treated beef. Arguably, the EU situation favours citizens, whilst the US policy favours agri-business. These differences have played out in the media particularly in the controversy over chlorine washed chicken [74,75]. However, they apply similarly to hormone treated beef, ractopamine pork and bovine somatotrophin use in dairy cattle [72].

## 8. Forms of Brexit and Animal Welfare

The form Brexit takes will have a substantial impact on whether threats and opportunities to animal protection materialise. This section discusses how a soft and hard Brexit might impact animal protection. A hard Brexit includes the possibility of a so-called ‘no deal’ Brexit, in which the UK and EU fail to agree on the Brexit terms. This means that trade between the UK and the EU and indeed between the UK and non-EU nations, would be on WTO terms.

### 8.1. A Soft Brexit

A soft Brexit means that the UK remains close to the EU in regulatory terms once it leaves the EU [69,76]. For our purposes, a soft Brexit might mean that the UK remains in either the single market and/or the customs union of the EU or negotiates a Free Trade Agreement meaning the same thing, with respect to agricultural goods. If the UK were to remain a member of the EU single market, it would retain regulatory alignment in agricultural policy, as well as a number of other policy areas that affect animal protection. If the UK were to remain in the customs union of the EU, it would retain the high EU tariffs on agricultural imports [77].

A soft Brexit therefore avoids the major risk of importing animal products that were raised and slaughtered in lower welfare standards compared to the EU/UK [10]. A soft Brexit leaves the UK to determine its own agricultural policy outside of the CAP [11]. However, continued membership of the single market would mean that the UK is not free to prohibit the live export of animals, or to ban the import and sale of goods produced in the EU such as foie gras. Table 2 in this paper highlights further policy changes based on different types of Brexit.

The above point about live animal transport and foie gras, however, should be considered in context. Simply because a soft Brexit permits the UK to prohibit live animal transport and the importation of foie gras does not mean that the government will implement these policies. Indeed, there will be significant political pressures to maintain the status quo. To illustrate, despite membership of the EU and therefore the single market precluding the prohibition of the importation of foie gras, nations that export the product, such as France, might challenge any UK prohibition at the WTO. The WTO is discussed in a later section.

### 8.2. A Hard Brexit

A hard Brexit means that the UK does not remain close to the EU in policy terms after leaving it [69,76]. For our purposes, a hard Brexit means that the UK leaves the single market and customs union of the EU. This means that the UK does not maintain regulatory alignment with the EU in agricultural and other policy areas. It also means that the UK is free to set its own rates on tariffs for imported goods, including animal products.

Like a soft Brexit, a hard Brexit also means that the UK can determine its own agricultural policy outside of the CAP [11]. At least in relation to being outside of the single market, it is not prevented from prohibiting the export of live animals and the prohibition of foie gras, although such a stance might be challenged at the WTO. A hard Brexit means that the UK is not a member of the customs union and can set its own tariffs. A hard Brexit is supported by many on the right of the Conservative Party in government because this enables the UK to set its own trade policy. However, given that high EU tariffs prevent the importation of goods produced to low welfare standards, a hard Brexit means there is a major risk of importing such goods and a race to the bottom in animal welfare [10,11,12].

### 8.3. Free Trade Agreements and WTO Rules

Post-Brexit, the UK government will aim to negotiate free trade deals with the EU and non-EU nations and trade blocs. The Department of Trade, led by the Atlanticist Liam Fox, has already launched consultations on free trade deals with the US, Australia and New Zealand [78]. Under free trade agreements, governments negotiate terms of the overall deal, including those related to animal welfare. Outside such bilateral and multilateral free trade agreements, nations that are members of the WTO are bound by its rules. The WTO is an intergovernmental organisational that exists to provide a framework for international trade and includes a dispute resolution system [79].

The WTO promotes free trade and its rules are concerned with preventing governments making policy to protect their own national industries at the expense of others. The UK has more progressive animal protection laws compared to most of the rest of the world. The more stringent regulation generally means it costs more for British farmers to produce meat and dairy products compared to international competitors with lower standards. For this reason, it is rational for the UK government to argue that animal welfare is a legitimate reason to restrict the importation of products produced to lower animal welfare standards. For instance, the UK government might insist that imports meet UK animal welfare standards, or it might erect protective tariffs for imports that do not [12]. If the UK does not do this, British farmers will be at a competitive disadvantage and may ultimately go out of business.

However, whether the WTO will view animal welfare as a legitimate reason to restrict trade is open to dispute. There is case law that does support animal welfare being considered as a legitimate reason to restrict imports [80]. These include the *US-shrimp case*, where the WTO Appellate Body ruled that it was permissible to condition market access based on methods with comparable effectiveness under WTO Article XX. Similarly, in the *EC-Seal Products* case, the Appellate Body of the WTO ruled that animal welfare is a legitimate issue of public morality in the EU [1]. Despite these cases, arguably there remains considerable uncertainty about how the WTO will rule on challenges to protection based on animal welfare.

## 9. Animal Protection Policy Making Inside and Outside of a Political Union

Given the UK’s decision to leave the EU, this paper has had three broad aims: (i) to provide a framework to investigate whether, all things considered, Brexit will be a net positive or negative for animal protection; (ii) to discuss the legal context of animal protection in the UK as a member state of the EU; and (iii) to discuss the political context of Brexit, which is key to investigating whether potential threats and opportunities related to Brexit and animal protection are likely to materialise.

Brexit is a major political event and the UK’s departure from the EU has the potential to have substantial and longstanding implications for animal protection. This is the case particularly because the UK is a relatively large nation in terms of population size and is an economically and politically powerful state. Furthermore, the UK has been a global leader in the area of animal protection, which is the object of our concern.

As a political event per se, Brexit therefore has enormous implications for animal protection. However, the EU-UK relationship and Brexit might serve as a useful case study to illustrate broader principles about animal protection policy making in a supranational body such as the EU versus that outside such a body. Indeed, the same or similar principles might apply to policy making in a federation of states, such as the US or Australia, compared to a non-federal state outside any supranational body, such as the UK post-Brexit.

As a member state of the EU, animal protection policy from the UK perspective is characterised by three considerations. First, as a powerful member state, the UK has leveraged its progressive animal protection stance to have a greater impact than it could otherwise have outside of the EU. The UK, with a human population of 67 million people, has positively influenced animal protection policy in the much larger EU of 510 million people. In effect the UK has leveraged its membership of the EU to impact animal welfare on a market seven to eight times the size of the UK itself. Indeed, the positive impact of the UK within the EU does not end there. The EU has itself used its status as the largest economic bloc in the world to influence animal protection internationally. Thus, as a member state of the EU, the UK has been able to multiply its progressive stance on animal protection to have a far greater impact than it could have achieved outside of the union.

Secondly, as a member state of the EU, the UK has in some cases been hampered in its more progressive stance on animal protection. The EU is composed of 28 member states that are involved in policy making in the various EU institutions. Whether voting is by qualified majority or by unanimity, less progressive EU members can hamper progressive reforms. This can leave some nations frustrated at either not being able to implement progressive reforms or being at a competitive disadvantage if they do so. In the EU, the member states in the north of Europe and in particular the UK, Sweden, Denmark and the Netherlands tend to be more progressive on animal protection. These nations sometimes implement EU directives earlier and/or with more stringent conditions compared to other EU member states.

Thirdly, the EU is a single market without borders for goods, people, services and capital. The rationale is to improve economic performance within the bloc by reducing barriers to trade. However, the single market also means that the UK as a member of the EU cannot restrict the importation of goods from the EU27 even in areas where it has higher standards of animal protection. For instance, the UK has effectively prohibited the production of foie gras on welfare grounds. Despite this, the UK is unable to prevent the importation and sale of foie gras from France and other EU nations, because of the single market.

We can call these three considerations first the leverage, secondly the inertia and thirdly the single market principles. The leverage and the inertia principle appear to be universal principles, in that they apply to any supranational body or federal nation that pool sovereignty to make common laws for economic and other advantages. In any supranational body or federal nation, the principle of leverage can be used either to reform or obstruct animal protection. The UK has used its membership of the EU to reform animal protection based on its progressive stance. However, a conservative or regressive nation or member, if sufficiently powerful, could employ the same leverage principle to obstruct such reforms. In contrast to the leverage principle, the inertia principle will, however, act to obstruct potential reforms that more progressive members lobby to have implemented. 

The single market principle does not appear to be universal. The single market is a key element of the EU, which considers the free movement of goods, capital, services and labour as sacrosanct. Despite this, the free movement of goods, for example agricultural products, is not a necessary condition for a supranational body, including a federation. For instance, in 2018 the US state of California has effectively prohibited the production *and* the importation of eggs produced in battery cages. California Proposition 12, the Farm Animal Confinement Initiative, establishes minimum space requirements for egg-laying hens. It also prohibits the importation of eggs into California produced from hens raised in confined conditions. The prohibition of the importation of eggs includes those produced in the other 49 states in the US. Proposition 12 also establishes minimum space requirements for calves raised for veal and breeding pigs [81].

These considerations about principles of policy making on animal protection both within supranational bodies and outside of them are key factors to consider when investigating whether Brexit is, all things considered, a net positive or negative for animal protection. The considerations also, however, have far wider relevance, in that they may be applicable in a broader sense outside of the EU-UK and Brexit context.

## 10. Conclusions

Brexit represents a major political upheaval for UK governance. It has the potential to have substantial impacts on animal protection policy not only in the UK but also in the EU and internationally. This paper has reviewed the relation between animal protection regulation in the EU and UK. A number of informed reports have been published on the threats and opportunities that Brexit presents. Whether these threats and opportunities materialise is dependent on a range of political factors. The paper therefore reviewed the political context of Brexit with a view to further research on whether the various threats and opportunities are likely to materialise.

The UK has been a member of the EU since 1993 and before that a member of the EC since 1973. Around 80 per cent of UK animal protection regulation is based on EU law. The European Union (Withdrawal) Act 2018 has nationalised the great majority of this law, although there are concerns about the powers the Act gives to ministers to amend legislation where relevant to make it appropriate after Brexit. Article 13 of the Treaty of Lisbon recognises animals as sentient beings and confers a duty on the EU and member states to pay full regard to animal welfare when formulating and implementing policy. Article 13 is part of the Treaty on the Functioning of the EU (TFEU) and therefore has not been carried over to UK law in the European Union (Withdrawal) Act 2018.

The UK referendum on EU membership was motivated by the Eurosceptic right wing of the Conservative Party and the rise of the UK Independence Party (UKIP). Since the referendum, Theresa May, who replaced David Cameron as Prime Minister, has appointed a number of right wing Eurosceptics to Cabinet to deliver Brexit. Key figures in the UK Government that will influence the impact of Brexit on animal protection are Theresa May, the Prime Minister, Michael Gove, the Defra Secretary and Liam Fox, the International Trade Secretary. The Conservative Government has made official policy statements to maintain and enhance animal protection after Brexit. 

However, the Conservative Party has a problematic relationship with animal protection. For instance, it faced a media furore after voting against a Green Party amendment to include Article 13 in the European Union (Withdrawal) Bill. Ultimately, the Government was forced to publish its own Bill, which contained a watered down version of Article 13. Similarly, the Conservative leadership faced widespread criticism over its decision to pledge a free vote on foxhunting in its 2017 general election manifesto. The Conservative Party has responded with a communications campaign to improve its image on animal welfare. The Government has published a number of consultations on animal protection issues.

The impact of Brexit on animal protection will be determined by its impact on farm animals. This is because of the far larger numbers of farm animals and also the reality that Brexit is more likely to impact animals used in agriculture more than experimental, wild and companion animals. Furthermore, the impact of Brexit on animal protection may be determined by its impact on animals in the EU and internationally. This is because there are far larger numbers of animals in the EU and internationally compared to the UK. For instance, whilst around 1 billion farm animals are raised and slaughtered in the UK annually, the figure for the EU is 4.7 billion and for the US 10 billion. 

A major threat of Brexit is the importation of lower animal welfare products, for instance from the US, after the UK leaves the EU. A major opportunity that Brexit presents is the potential for major reform of UK agricultural policy based on a subsidy system focused on rewarding animal welfare as a public good. Softer forms of Brexit, in which the UK remains in the single market and/or the customs union of the EU or their equivalents, mitigate this major threat. A soft Brexit, with respect to agriculture, makes it very unlikely that the UK will import lower welfare animal products. Theresa May’s Chequers agreement, which established the policy of the UK Government on Brexit in June 2018, is effectively a soft Brexit for agriculture and therefore effectively for animal protection. Trade in goods, including agricultural goods, would be governed according to a ‘common rule book.’ This effectively would mean a continuation of the single market for agriculture.

The potential for Brexit having an international impact on animal welfare was demonstrated during the visit of the US President, Donald Trump, to the UK. Trump was publicly critical of May’s Chequers agreement soft Brexit. The US is a major exporter of agricultural products such as beef. Based on trade considerations, a hard Brexit is far more favourable to Trump and the US, than a soft Brexit where the UK continues regulatory alignment with the EU.

Brexit is highly unstable and its impacts on animal protection are uncertain. This paper has provided an overview of the EU and UK regulatory relationship on animal protection to provide a baseline of the status quo. Whether the various threats and opportunities that Brexit presents will materialise depends on a range of political factors. This paper has discussed the political context of Brexit to inform that issue, which has the potential to affect the welfare of billions of animals in the UK, the EU and internationally. Further research is needed to assess if the major threats and opportunities related to Brexit are likely to materialise.

## Figures and Tables

**Table 1:** Numbers of animals by category in the UK, EU and US.

Category of Animal	Species	EU 28 (incl.UK)	UK	US
Agriculture	Includes poultry, pigs, cattle and sheep ^1^	4.7 billion [13]	1 billion [14]	10 billion [15]
Research		11 million [16]	4 million (procedures) [17]	12-27 million ^2^ [18]
Wild ^3^		Unknown	Unknown	Unknown
Companion	Fish	-	51 million ^4^	58 million [19]
	Dogs	65 million [16]	9 million	90 million [20]
	Cats	99 million [16]	8 million	94 million [20]
	Horses	7 million [21]	1 million	7.6 million [20]

^1^ The majority of agricultural animals raised and killed are poultry, specifically chickens; ^2^ US figures are imprecise as fish and birds are not officially recorded; ^3^ No figures for wild animals; ^4^ UK figure for companion animals includes aquaria and ponds.

**Table 2:** Animal welfare and Brexit scenarios. Reproduced with permission from the Brexit & Animals Taskforce [11].

Animal Welfare Issue	Model of Relation with the EU27					
	EU	EEA ^1^	CH ^2^	CU ^3^	DCFTA ^4^	WTO ^5^
Ban live exports for fattening and slaughter	✕	✕	✓	✕	✓	✓
Introduce method of production labelling	✕	✕	✕	✓	✓	✓
Introduce new farm support system to encourage high level of animal welfare	✕	✓	✓	✓	✓	✓
Participate in PETS	✓	✓	✓	✓	✓	✓
Additional veterinary requirements for dogs and cats moved via non-commercial means into UK	✕	✕	✓	✕	✓	✓
Maintain ban on testing of cosmetics products and ingredients on animals and marketing ban on animal-tested cosmetics from outside UK and EU	✓	✓	✓	✓	✓	✓
Continued collaboration with key EU regulatory agencies/enforcement bodies	✓	✓	✕	✕	?	✕
Continued full access to TRACES (tracking of shipment and use within the territory)	✓	✕	✕	✕	✕	✕
Tariff-free access for agricultural goods	✓	✕	?	✕	?	✕
Tariff-free access to veterinary medicines	✓	✓	✓	✓	?	✕
Unimpeded movement of animals between the EU and the UK	✓	✕	✕	✕	✕	✕

^1^ Norway model; ^2^ Swiss model; ^3^ Customs Union; ^4^ Deep and comprehensive free trade agreement; ^5^ World Trade Organisation.

**Table 3:** Key EU animal protection regulation.

Category	EU Law
Farmed animals	Directive 98/58/EC concerns the protection of animals kept for farming purposes
	Regulation (EC) No. 1/2005 on the protection of animals during transport and related operations
	Regulation (EC) No. 1099/2009 on the protection of animals at the time of killing
	Directive 1999/74/EC laying down minimum standards for the protection of laying hens
	Directive 2007/43/EC laying down minimum rules for the protection of chickens kept for meat production
	Directive 2008/119/EC laying down minimum standards for the protection of calves
	Directive 2008/120/EC laying down minimum standards for the protection of pigs
Companion animals	Regulation (EU) No 576/2013 of the European Parliament and of the Council of 12 June 2013 on the non-commercial movement of pet animals and repealing Regulation (EC) No 998/2003
Research animals	Directive 2010/63/EU of the European Parliament and of the Council of 22 September 2010 on the protection of animals used for scientific purposes
	Regulation (EC) No 1907/2006 of the European Parliament and of the Council of 18 December 2006 concerning the Registration, Evaluation, Authorisation and Restriction of Chemicals (REACH), establishing a European Chemicals Agency
Wild animals	The Convention on the Conservation of European Wildlife and Natural Habitats
	The Convention on the Conservation of Migratory Species of Wild Animals
	Council Directive 92/43/EEC of 21 May 1992 on the conservation of natural habitats and of wild fauna and flora
	Directive 2009/147/EC of the European Parliament and of the Council of 30 November 2009 on the conservation of wild birds
	Regulation (EU) No 1143/2014 of the European Parliament and of the Council of 22 October 2014 on the prevention and management of the introduction and spread of invasive alien species
